# Is Higher Consumption of Animal Flesh Foods Associated with Better Iron Status among Adults in Developed Countries? A Systematic Review

**DOI:** 10.3390/nu8020089

**Published:** 2016-02-16

**Authors:** Jacklyn Jackson, Rebecca Williams, Mark McEvoy, Lesley MacDonald-Wicks, Amanda Patterson

**Affiliations:** 1 School of Health Sciences, Faculty of Health and Medicine, University of Newcastle, University Drive, Callaghan, NSW 2308, Australia; Jacklyn.Jackson@uon.edu.au (J.J.); Lesley.Wicks@newcastle.edu.au (L.M.-W.); 2 School of Biomedical Sciences and Pharmacy, Faculty of Health and Medicine, University of Newcastle, University Drive, Callaghan, NSW 2308, Australia; Rebecca.Williams@newcastle.edu.au; 3 Centre for Clinical Epidemiology and Biostatistics, Hunter Medical Research Institute, University of Newcastle, Callaghan, NSW 2308, Australia; Mark.McEvoy@newcastle.edu.au; 4 Priority Research Centre in Physical Activity and Nutrition, University of Newcastle, NSW 2308, Australia

**Keywords:** systematic review, animal flesh, iron status, adults, developed countries

## Abstract

Iron deficiency (ID) is the most prevalent nutrient deficiency within the developed world. This is of concern as ID has been shown to affect immunity, thermoregulation, work performance and cognition. Animal flesh foods provide the richest and most bioavailable source of dietary (haem) iron, however, it is unclear whether low animal flesh diets contribute to ID. This systematic review aimed to investigate whether a higher consumption of animal flesh foods is associated with better iron status in adults. CINAHL, Cochrane, EMBASE and MEDLINE were searched for published studies that included adults (≥18 years) from developed countries and measured flesh intakes in relation to iron status indices. Eight experimental and 41 observational studies met the inclusion criteria. Generally, studies varied in population and study designs and results were conflicting. Of the seven high quality studies, five showed a positive association between animal flesh intake (85–300 g/day) and iron status. However, the optimum quantity or frequency of flesh intake required to maintain or achieve a healthy iron status remains unclear. Results show a promising relationship between animal flesh intake and iron status, however, additional longitudinal and experimental studies are required to confirm this relationship and determine optimal intakes to reduce ID development.

## 1. Introduction

Iron deficiency (ID) is the most prevalent nutrient deficiency worldwide, affecting over 30% of the population [1,2]. This is a major concern as iron in the human body contributes to many important physiological functions including oxygen transport, energy production and neurotransmitter synthesis [1,3,4,5]. Further, the health consequences associated with ID anaemia such as impaired growth and development, reduced cognitive function, adverse pregnancy outcomes, poor immunity and thermoregulation have long been recognized [1,4,5]. There is building evidence to suggest that ID without anaemia is also linked to reduced capacity for physical activity, reduced productivity and increased fatigue leading to poorer cognitive work performance [4,6].

Within developed countries, ID remains the most widespread nutrient deficiency, and is common among specific population groups such as infants and young children, adolescents, women of childbearing age, the elderly and those from socially disadvantaged and Indigenous backgrounds [1,4,7,8,9,10]. Data from the National Australian Health Survey from 2011 to 2012 supports this notion as approximately 760,000 Australian adults (4.5%) were found to be at risk of iron deficiency anaemia, with women at greater risk than men (6.4% compared with 2.5%) [11]. Further, the risk of anaemia was highest among older Australians, with rates of anaemia rapidly increasing at the age of 65 years [11]. Other common lifestyle factors that have been shown to increase the risk of developing ID include high endurance physical activity, consumption of a diet low in bioavailable dietary iron and blood donation [4].

ID occurs when iron intake or absorption is insufficient to meet body iron requirements or increased iron demands [4]. ID is a dynamic process and is characterized by a reduction in stored iron, most frequently measured by the blood marker serum ferritin [1]. However, the determinants of iron absorption are complex and depend on multiple factors, including the individual’s iron status, as well as the iron content and composition of the meal [1,3,4,5,12]. There are two types of dietary iron: haem iron from animal flesh foods and non-haem iron, which is the only type found in plant based foods such as grains and vegetables [3,4]. In humans, the haem form is the most bioavailable, with an estimated absorption rate of 11%–22% [12,13]. This is much higher than non-haem iron, with an estimated absorption rate of 1%–7% [13]. Thus, it has been established that total dietary iron intake does not always correlate with biochemical iron status [13,14]. The dietary content of iron absorption inhibitors and enhancers are another important consideration in determining iron status as they act to increase or reduce iron absorption, mostly non-haem iron [4]. The main inhibitors of non-haem iron absorption include calcium, phytates in high fibre foods and phenolic compounds from tea and coffee. The main enhancers include ascorbic acid and un-identified factors found in meat while other factors such as alcohol can also assist iron absorption [3,4,5,12,13].

Animal flesh foods (such as beef, pork, lamb, poultry and fish) provide a rich source of dietary protein, iron, iodine, zinc, Vitamin B_12_ and omega-3 fatty acids [15,16,17,18]. In Australia, it is recommended that adults consume a maximum of 65 g of lean meat/day (455 g/week) as part of a healthy and nutritionally balanced diet [15,16,17,18]. In recent times, the use of dietary intervention for the treatment of mild ID was advocated [19], a notion that has been supported in a study conducted by Patterson *et al.* [19] which investigated the effect of improving iron status, with a high bioavailable diet, on general health, wellbeing and tiredness within ID women aged 18–50 years. In general, these women experienced improved mental health and vitality as iron status improved [19]. The study also compared supplement and dietary treatment for ID and found that, despite greater iron status improvements seen in the supplementation group, the diet group experienced greater improvements in health and wellbeing, thus indicating the very important role of dietary intervention for the treatment of ID [19].

Dietary modification to include greater quantities of animal flesh foods is theoretically a relatively simple intervention, however, the true impact this intervention could have on iron status is not clear. To the authors’ knowledge, this is the first systematic review conducted with the purpose of determining if a higher consumption of animal flesh foods results in better iron status for adults within developed countries. This is a highly important and topical question given the recent health, environmental and ethical concerns associated with animal flesh consumption, which have prompted recommendations for the population to reduce their consumption of animal flesh foods [20,21]. It is important to note, however, that animal flesh foods remain the richest source of bioavailable dietary iron, and the effect reduced intakes could have on the population’s iron status is not known [21,22].

## 2. Methods

### 2.1. Inclusion Criteria

#### 2.1.1. Types of Participants

This review considered studies that included both male and female adults, aged 18 years and older. Only studies conducted on populations within developed countries were included, as ID risk in populations from underdeveloped countries is especially high due to prevalence of malnutrition and infectious diseases [4]. Currently, there is no established convention for the definition of a developed country. Thus, for the purpose of this review, countries were identified and included as developed countries based on the member countries of the Organisation for Economic Co-operation and Development (OECD). These include Australia, Austria, Belgium, Canada, Czech Republic, Demark, Finland, France, Germany, Greece, Iceland, Ireland, Israel, Italy, Japan, Korea, Luxembourg, Netherlands, New Zealand, Norway, Poland, Portugal, Slovak Republic, Slovenia, Spain, Sweden, Switzerland, United Kingdom and the United States.

#### 2.1.2. Types of Studies

Study types included for review were experimental studies such as: Randomised Controlled Trials (RCT); quasi-RCTs; matched pairs RCTs; cross over trials; and pre and post studies. Observational studies such as cross sectional studies, prospective and retrospective cohort studies and case control studies were also considered. Included studies were those assessing iron status measures (such as serum ferritin and haemoglobin) in relation to dietary intake of animal flesh foods. Animal flesh foods were defined as: the muscle tissue of an animal carcass. This included red and white meats derived from chicken/poultry, sheep, pig, cattle, goat, fish, seafood, buffalo, kangaroo, camel, deer or rabbit. This definition includes the muscle component only and excludes offal such as liver and kidneys. For the purpose of this review, processed meats such as ham, bacon, and sausage were also included.

#### 2.1.3. Types of Outcomes

Studies were considered for review if iron status was measured as an outcome identified by measurement of iron status indices in the blood, or participants were classified as having normal iron concentrations, iron depletion, iron deficient erythropoiesis or iron deficiency anaemia. Additionally, studies using self-reported ID outcomes, such as “diagnosis of iron deficiency by a medical professional” were also considered.

### 2.2. Search Strategy

The aim of the search strategy was to find published studies in the English language dated up until October 2015. A systematic literature search of four electronic databases (CINAHL [23], Cochrane [24], EMBASE [25] and MEDLINE [26]) was undertaken with the help of an academic librarian using key search terms (see Supplemental Table S1). Based on the articles title, abstract and description, the relevance of each study was evaluated by two independent reviewers. Studies identified as meeting the selection criteria, or those that were unclear, had the full article retrieved. The full-text articles were assessed by two independent reviewers, and a third reviewer was consulted when the first two disagreed. The reason for a study’s exclusion from the review was recorded.

### 2.3. Study Quality

Included studies were assessed for methodological quality using the Academy of Nutrition and Dietetics (AND) Quality Criteria Checklist [27], to identify the strength of research design, relevance and validity. Sources of bias, such as population selection and blinding, were also considered using the AND Quality Criteria Checklist. Two independent reviewers used the checklist to assign an overall quality rating to the study as positive, neutral or negative, and a third reviewer was consulted if there was a discrepancy.

### 2.4. Data Extraction and Synthesis

Data extraction was undertaken by one reviewer and checked for accuracy and consistency by a second reviewer. Participant and intervention characteristics were extracted as well as data in relation to review outcomes. Due to the heterogeneity of the included studies, meta-analysis was not performed. Therefore, the effect of animal flesh food intake on iron status was described in a narrative synthesis. Data synthesis was conducted to estimate the direction of effect, the strength of the evidence for the effect, and to assess whether the effect was consistent across studies.

## 3. Results

### 3.1. Study Selection

**Figure 1:**
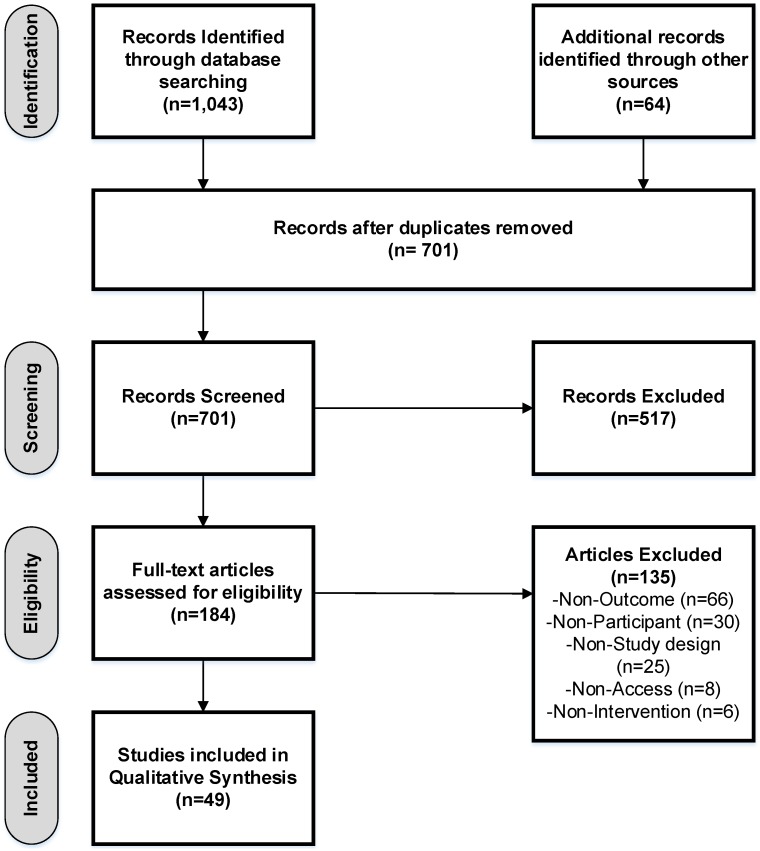
PRISMA flow diagram of study selection process.

Of the 1107 abstracts identified (1043 from database search and 64 from reference list checking), 184 full-text articles were retrieved. Forty-nine studies [14,28,29,30,31,32,33,34,35,36,37,38,39,40,41,42,43,44,45,46,47,48,49,50,51,52,53,54,55,56,57,58,59,60,61,62,63,64,65,66,67,68,69,70,71,72,73,74,75] met the inclusion criteria and were included in this review (Figure 1. PRISMA (Preferred Reporting Items for Systematic Reviews and Meta-Analyses) flow diagram).

### 3.2. Study Characteristics

The total number of participants across all included studies was 111,846 (mean *n* = 2283; range 14–72,833). Of the total number of participants, only 9.6% were male. Two studies [70,71] included exclusively all-male populations, compared with 29 studies [14,29,30,31,32,33,34,36,37,42,45,46,48,50,51,52,53,54,56,58,60,61,62,64,67,68,73,74,75] that included exclusively females. The remaining 18 studies included mixed gender populations [28,35,38,39,40,41,43,44,47,49,55,57,59,63,65,66,69,72], to which females frequently contributed greater than 50% of the study population. The age of participants across all included studies ranged from 18 to 93 years. While 36 studies included adult participants ≥18 years of age, six studies [29,33,34,37,48,54] included exclusively young adult participants (18–35 years) and seven studies [38,39,40,41,59,66,69,70] included only elderly participants (≥65 years). Most of the studies’ participants were of apparently good health, however, 11 studies [14,36,37,45,52,56,60,61,66,68,72] reported presence of various diseases (cardiovascular disease, cancer, diabetes, liver disease) and conditions (overweight, obesity, eating disorders, iron deficiency). Eighteen studies were conducted in the United States [34,40,41,43,48,49,50,51,52,55,56,58,67,68,70,73,74,75] and seven in Australia [30,37,47,54,60,61,71]. The remaining studies were conducted in the United Kingdom (*n* = 4) [36,45,64], New Zealand (*n* = 5) [28,31,32,44,46], Denmark (*n* = 3) [14,59,63], the Netherlands (*n* = 2) [35,38], Spain (*n* = 2) [33,69], with one each in Belgium [62], Canada [57], France [42], Germany [72], Israel [55], Japan [29], South Korea [53] and Switzerland [65] (See Supplemental Table S2).

Only eight of the included studies were experimental in design, with interventions ranging from eight weeks to 12 months’ duration [14,34,37,50,51,58,60,70]. The remaining studies were either cross-sectional [28,29,30,31,32,33,35,38,39,40,41,43,44,45,46,47,48,49,52,53,54,55,56,57,59,61,65,66,67,69,71,73,74,75] or prospective cohort studies [36,42,62,63,64,68,72]. Of the experimental studies, one focused on manipulating the amount and type of meat consumed for lunch and monitoring its effect on iron status in young women [34]. Three studies [37,50,60] compared the iron status of women consuming a high meat diet with a low meat diet, however in two of these studies, participants were also consuming iso-caloric diets, as weight loss was the primary outcome for the studies [37,60]. Three studies [14,51,70] compared the effect of consuming a meat containing diet with a vegetarian diet on iron status. Additionally, one study [58] investigated the effect of consuming meat during an exercise intervention on iron status with a “no intervention” control and placebo group (exercise and placebo capsule).

Of the 41 observational studies, 14 [28,30,38,43,44,47,49,53,55,57,62,64,67,71] compared iron status of participants consuming vegetarian and omnivore diets, while three studies [36,45,73] compared the iron status of red meat eaters, fish and/or poultry eaters and vegetarians. Eight studies [31,32,35,39,54,59,61,69] investigated the correlation between iron status and total animal flesh intake. Additionally, 12 studies [29,33,40,41,42,46,52,56,63,66,74] investigated the correlation between iron status and intakes of different types of animal flesh (red meat: unprocessed and/or processed meats, white meat: poultry and/or fish) separately, while four studies investigated the correlation between red meat [65,68,72] or beef [75] intake and iron status.

The most common iron status indicators reported across the included studies were serum ferritin (SF), Haemoglobin (Hb) and serum iron. Additionally, because measures of iron status may be affected by age, gender, dehydration, exercise, inflammation or malignancy [4], some of the studies also measured C-Reactive Protein (CRP), Total Iron Binding Capacity (TIBC), serum Transferrin (sTf), Transferrin saturation (Tf saturation) and serum Transferrin Receptor (sTfR), to prevent misdiagnosis of iron status [4].

### 3.3. Risk of Bias within Studies

Results from the Academy of Nutrition and Dietetics (AND) Quality Criteria Checklist [27] revealed that only seven studies [14,34,46,60,62,66,74] out of 49 studies were of a positive quality rating. However, of these positively rated studies, one experimental study [60] and four observational studies [46,62,66,74] did not clearly blind participants or data collectors. Another experimental study [14] had not clearly accounted for participant withdrawals or clearly indicated sources of study funding or conflicts of interest. Four studies [35,47,55,57] were identified as negative in quality rating, as the main criteria for participant selection bias, generalisability, study methodology and analysis were commonly not met. The remaining 38 studies [28,29,30,31,32,33,36,37,38,39,40,41,42,43,44,45,48,49,50,51,52,53,54,56,58,59,61,63,64,65,67,68,69,70,71,72,73,75] were of neutral quality, an outcome most frequently influenced by bias of study participant selection and lack of participant and data collector blinding to study outcomes (Refer Supplementary Table S3).

### 3.4. Effect of Animal Flesh Intake on Iron Status in Adults within Developed Countries

#### 3.4.1. Experimental Studies

Of the experimental studies investigating the effects of a high red meat diet (~300 g lean meat/day) compared to a low red meat diet (~80 g lean meat/day) on iron status in women, Cheng *et al*. [37] and Noakes *et al.* [60] found serum ferritin (SF) levels were significantly (*p* = 0.021 [37], *p* = 0.004 [60]) greater in the high red meat group at the end of the intervention periods ranging from 12 weeks [60] to 12 months [37]; however, a cross-over trial by Hunt *et al.* [50] found in post-menopausal women SF and other blood indices were significantly (*p* = 0.01) higher in the low meat diet group (38.5 g/day meat) compared with the high meat group (289 g/day meat) at the end of the seven week intervention period. Similarly, in an eight weeks experimental study conducted by Hunt *et al.* [51], none of the blood indices for iron status were altered by the consumption of an omnivorous diet compared with a vegetarian diet in postmenopausal women. Yet a different 20 weeks experimental study by Tetens *et al.* [14] found that in young women, a meat based diet was able to maintain SF and Hb levels during the diet intervention period compared with the vegetarian based diet group that experienced a significant reduction in SF (*p* < 0.001) and Hb (*p* = 0.003) levels.

Wells *et al.* [70] compared the effect of a beef based diet with a vegetarian based diet in exercising elderly males; in which the beef based diet led to increased Hb and haematocrit (Hct) levels, but remained stable in the vegetarian group. SF levels however were reduced in both diet groups over the 12 weeks intervention period. Lyle *et al.* [58] investigated the effect of a meat based diet during an exercise intervention among females, and found contrasting results, as the meat based group displayed greater SF values than the placebo group and significantly (*p* < 0.02) greater SF values than the control group. In a 16 weeks randomised controlled trial by Blanton [34], the amount and type of meat consumed for lunch was manipulated. It was found that in young women, the beef lunch group (85 g beef) led to significantly (*p* < 0.0001) greater SF and Hb concentrations than the non-beef lunch group (85 g, non-beef) at the end of the intervention [34]. However, the number of SF responders was non-significant between groups [34]. (See Table 1. For the results of the experimental studies). 

#### 3.4.2. Observational Studies Comparing Omnivorous Diets with Vegetarian Diets

Of the 13 vegetarian and omnivore comparisons, nine studies [28,30,47,49,53,54,64,67,71] found SF concentrations to be significantly higher in the omnivore participants compared with the vegetarian participants, with four additional studies [38,44,55,57] reporting that omnivores had higher SF concentrations than their vegetarian counterparts, however, none of the four studies reached statistical significance. Three more studies [47,64,67] identified vegetarian populations to be at significantly greater risk of ID than omnivores, a relationship which Snyder *et al.* [67] found apparent once iron supplement users were excluded from the analyses. Alexander *et al.* [28] and Haddad *et al.* [43] found SF concentrations to be significantly higher in omnivore males compared with vegetarian males, however, both studies found iron stores were consistently low across female populations regardless of diet group. While Ball *et al.* [30] found that omnivores had greater SF concentrations than vegetarians, the higher SF concentration correlated weakly with daily meat consumption, however, this was not statistically significant. (See Table 2. For results of the observational studies).

**Table 1:** Experimental results: Intake of animal flesh foods and iron status.

Study	Population (Gender, Age)	Intervention Group/s	Comparator/s	Iron Status Measures	Results	Limitations
Blanton. (2014) [34]	Female, 18–30 years	Beef lunch group: 4 weeks cyclic menu of 85 g beef per day for 16 weeks. Consume ≤1 serve of beef outside study. Beef = eye round, roast, top sirloin, ground beef 90% lean.	Non-beef lunch group: 4 weeks cyclic menu of 85 g of non-beef per day for 16 weeks. Consume ≤1 serve of beef outside of study. Non-Beef = egg, chicken, turkey, cheddar, Swiss cheese, pork, ham.	Fasting blood samples at baseline, week 8 & 17. Hb, Hct, MVC, MCH, MCHC, sFe, TfR, Tf, Tf saturation, SF	At week 16: (Mean ± SD) **SF (ng/mL):** Beef: 41 ± 6.8 Non-Beef: 29.2 ± 3.0 (*p* < 0.0001) **TfR (mg/L):** Beef: 3.2 ± 0.2 Non-Beef: 3.4 ± 0.2 (*p* < 0.0001) **Hb (g/L):** Beef: 146.2 ± 02 Non-Beef: 140.7 ± 0.2 (*p* < 0.0001)	Homogenous population and possible sample bias. Unable to strictly control intakes of free-living participants. Participants not blinded to diet group allocation.
Cheng *et al.* (2013) [37]	Female, 18–25 years	High Protein (HP) group: Iso-energy diet for 12 mo. Consumed 300 g (raw) meat (Lean white or red meat)/day.	High Carbohydrate (HC) group: Iso-energy diet for 12 mo. Consume 80 g (raw) meat/d.	Fasting blood sample collected at baseline, 6 mo and 12 mo. Hb, sFe, Tf saturation, SF and CRP.	12 mo. from baseline: **SF (ng/L):** HP: 52.0 µg HC: 39.0 µg (*p* = 0.021) **sTfR-index:** HP: 0.89 HC: 1.05 (*p* = 0.024) **Hb (g/L):** HP: 132 ± 12 HC: 126 ± 11 (*p* = 0.075)	Use of contractive medications was a confounder. Low retention rate.
Hunt *et al.* (1999) [51]	Female, 20–42 years	Non-vegetarian group, consumed approximately 184 g meat/day.	Vegetarian group, consumed 0 g of meat/day.	Fasting blood samples after week 7–8. Measured: Hb. sFe, TIBC, Tf saturation, SF and CRP	After 8 weeks from baseline, blood indices were **NS** between the groups.	Diet groups may contain confounding by presence of inhibitors and enhancers.
Hunt *et al.* (1995) [50]	Female, 51–70 years	High Meat (HM) group, consume 289 g of meat/day.	Low Meat (LM) group, consume 38.5 g of meat/day.	Blood samples after week 7. Measured: Hb, Hct, sFe, IBC Tf saturation, sTf, SF	7 weeks from baseline: **SF (ng/L):** HM: 74 LM: 82 (*p* = 0.01) **IBC (nmol/L):** HM: 58 LM: 53 (*p* = 0.002) **Tf saturation (%):** HM: 27.2 LM: 31.4 (*p* = 0.03)	Blood samples were non-fasting. CRP was not measured. No wash out period between diet groups.
Lyle *et al.* (1992) [58]	Female, >18 years	Meat and Exercise (M + Ex) = High iron diet including low fat muscle meat foods + exercise.	Control group = free choice diet and no exercise. Placebo and exercise group = placebo capsule per day + free choice diet + exercise	Blood sample at week 4, 8 and 12. Measured: sFe, Total IBC, SF, Tf saturation, Hb and Hct	At 12 weeks from baseline **Hb (g/L):** M + Ex = 50.2 ± 8.7 (*p* < 0.05, increase) P + Ex = 51 ± 8.6 (*p* < 0.05, reduction) Control = 46.6 ± 8.7 (NS, stable) **SF (ng/L):** M + Ex = 29.2 ± 16.0 (NS, increase) P + Ex = 23.9 ± 15.5 (*p* < 0.05, reduction) Control = 12.7 ± 12.6 (*p* < 0.05, reduction)	Study could not blind participants to the meat and exercise group. Unable to separate the effect of the diet and the exercise.
Noakes *et al.* (2005) [60]	Female, 20–65 years	High Protein (HP) of 5600 kJ/day, low in saturated fat. Include ≥200 g lean beef or lamb >6 times/week and extra 100 g chicken or fish daily.	High Carbohydrate (HC) diet of 5600 kJ/day, low is saturated fat. Include 80 g chicken, pork or fish >6 times/week and red meat <1/week.	Fasting blood at baseline, week 4, 8 and 12 to measure: Hb and SF	At end of intervention: **Hb:** HP: 2% increase (*p* = 0.116) HC: NS **SF (ng/L):** Fe replete participants were excluded: HP: 120 ± 17 (41% increase) HC: 90 ± 12 (NS)	It is unclear if infection or other disease states influenced higher SF values as CRP was not measured.
Tetens *et al.* (2007) [14]	Female, 19–39 years	Meat diet group consumed 152 g (147–168 g) of meat (pork, beef or chicken) per day.	Vegetable diet group consumed 31 g (24–36 g) of meat/day.	Fasting blood collected at baseline, week 10 and 20. To measure: SF and Hb.	Baseline *vs.* week 20. **SF (ng/L):** Meat: 16.3 *vs.* 16.5 (*p* = 0.071) Vegetable: 17.3 *vs.* 11.2 ( *p* < 0.001) **Hb (g/L):** Meat: 126 ± 0.9 *vs.* 125 ± 1.1 (NS) Vegetable: 124 ± 0.9 *vs.* 121 ± 0.9 (*p* = 0.003)	CRP was not measured. Vegetarian diet contains inhibitors likely to reduced iron absorption and thus iron status.
Wells *et al.* (2003) [70]	Male, 59–78 years	Beef group: beef containing diet with resistive training 3 weeks.	Vegetarian group: Texturised vegetables protein. e.g., veggie and chickpea patties and veggie dogs. Resistive training 3/week.	Fasting blood samples collected at baseline, week 5 and 12. Measured: Hb, Hct, MCV, MCH, sFe, Total IBC, TfR and SF.	Baseline *vs.* end of intervention: **Hb (g/L):** Beef: 140 ± 6 *vs.* 151 ± 9 Veg: 143 ± 7 *vs.* 145 ± 7 (*p* < 0.01, group × time interaction) **SF (µg/L):** Beef: 132 ± 107 *vs.* 131 ± 132 Veg: 95 ± 70 *vs.* 72 ± 53 (*p* < 0.01, time effect)	Small sample size.

FFQ = Food Frequency Questionnaire; Hb = Haemoglobin; MCV = Mean Corpuscular Volume; MCH = Mean Corpuscular Haemoglobin; MCHC = Mean Corpuscular Haemoglobin Concentration; sFe = Serum Iron; TfR = Transferrin Receptor; Tf = Transferrin; SF = Serum Ferritin; Fe = Iron; CRP = C-Reactive Protein; Kj/day = Kilojoules per day; NS = Not Significant; IBC = Iron Binding Capacity.

#### 3.4.3. Observational Studies Comparing Iron Status Based on Flesh Intake

Of the three studies [36,45,73] investigating the iron status of participants based on their red meat, fish and poultry intake, Cade *et al.* [36] reported that red meat and poultry consumption was positively associated with SF, but that fish was not; further those who consumed red meat daily had 36% higher SF concentrations than non-red meat consumers. Superior iron status for heavy red meat consumers (>5 serves red meat/week) was also found by Worthington-Roberts *et al.* [73] yet the iron stores of omnivores consuming fish and poultry (>5 serves fish or poultry/week and ≤1 serve red meat/week) were lower than that of the vegetarian participants. Contrasting results were found by Harvey *et al.* [45], who reported significantly (*p* < 0.01) greater median SF concentrations for the poultry and fish group (450 g poultry and fish and 90 g pork/week) than the red meat group (minimum 450 g red meat/week). The red meat group also had median SF concentrations lower than the vegetarian group, and in addition, the red meat and vegetarian groups displayed greater mean sTfR concentrations (poorer iron status) than the poultry and fish group [45].

Six other observational studies [42,46,52,54,63,75] found a positive correlation between total flesh meat consumption and iron stores. Heath *et al.* [46], found that women with mild ID had a mean flesh intake of 86 g/day compared with 111 g/day in women without mild ID. Additionally, Kato and colleagues [52] also found that women consuming >104.5 g/day of meat had 30% greater SF levels than those consuming only 42.4 g/day. Further, Kato *et al.* [52] and Galan *et al.* [42] found this trend was more pronounced with the consumption of red meat rather than white meat. Whereas, Rigas *et al.* [63] found the consumption of fish to be positively associated with the iron stores of male participants only. Similar findings have been repeated in a number of other studies [33,40,41,72,74] that reported that red meat (sometimes including processed meat) was a significant predictor of SF and iron stores, but that poultry and fish intake were not. Blanco-Rojo and colleagues [33] clearly demonstrated this, showing a significantly (*p* < 0.05) higher consumption of red meat in iron replete women (75.8 ± 66.0 g/day) than in women with mild ID (35.5 ± 40.4 g/day), a relationship that was not statistically significant for other animal flesh foods. Fleming and colleagues [40] also found that for each additional serving of red meat (113.6–170 g) per week, there was a 6% increase is SF values. Furthermore, studies [65,68] have found a higher consumption of red meat to be marginally associated with a lower risk of poor iron status. Ley *et al.* [56] found total red meat and poultry intake were positively associated with SF, however, this association failed to reach significance. Askakura *et al.* [29] found no association between the risk of developing ID and intake of meat and fish. Houston *et al.* [48] reported a correlation between frequency of red meat consumption and Hb levels, however, SF levels were higher in red meat abstainers, but to a non-statistically significant degree.

A study by Patterson *et al.* [61] found mean intakes of meat, fish and poultry in groups of ID (90.3 g ± 56.5) and iron replete (104.7 g ± 45.9) women to be non-statistically different. Doyle *et al.* [39] found a positive relationship with the intake of meat, poultry and fish in men and women with Hb concentrations, but not for SF concentrations, a finding consistent with Pynaert *et al.* [62]. Yet Milman *et al.* [59] found a positive correlation between SF and meat intake for men, but not women. However, intake of meat was found to be positively correlated with SF levels in male and female participants in two other studies [35,69]. Yet Beck *et al.* [32] found that participants consuming the greatest quantities of meat and vegetables displayed significantly greater SF concentrations, than those consuming the least. Further, Beck *et al.* [32] reported that a higher consumption of a meat and vegetable based diet reduced the risk of developing ID by 41%. An additional study by Beck *et al.* [31] produced similar results, however, once other variables were adjusted for, the effect was only significant in women with children. (See Table 2. For results of the observational studies). 

**Table 2:** Observational studies results: Intake of animal flesh foods and iron status.

Study	Population (Gender, Age)	Animal Flesh Definition	Dietary Data Collection Tool	Iron Status Measures	Results	Limitations
Alexander *et al.* (1994) [28]	Male (27%) and Female, >18 years	Vegetarian = no red meat and chicken, but may consume fish <1/week. Vegans = no animal flesh. Omnivore = no restriction of flesh intake	12-day semi—quantitative food record. Photographs of representative portion sizes of common foods difficult to directly estimate were included.	Blood collected to estimate: SF and Hb.	**SF (ng/L):** Men Veg = 36.6 Men Omn = 105.4 (*p* < 0.01) Women Veg = 13.6 Women Omn = 33.6 (*p* < 0.01) **Males with SF <12 ng/L** Veg = 29% Omn = 7% (*p* < 0.01)	Non-representative sample. Exclusion of participants using iron supplement was inconsistent. Unclear if blood samples were fasting. CRP not measured.
Askakura *et al.* (2009) [29]	Female, 18–25 years	Meat and fish (not otherwise defined)	Validated, self-administered diet history questionnaire.	Blood samples, measured: sFe, SF and Hb	Meat intake of Q1 = 16 g/day, Q5 = 54 g/day **Prevalence of ID based on meat intake:** Q1 = 52/151 Q5 = 46/158 (NS, *p* = 0.17) Fish intake of Q1 = 11.6 g/day, Q5 = 48 g/day **Prevalence of ID based on fish intake:** Q1 = 50/153 Q5 = 50/154 (NS, *p* = 0.94)	Non-representative sample. Dietary intakes based on self-reported data. Exclusion of participants based on use of iron supplementation was inconsistent. Unclear if blood samples were fasting.
Ball *et al.* (1999) [30]	Female, 18–45 years	Vegetarian = eat red meat <1/mo, and consume chicken or fish <1 week for >6 mo. Omnivore = eat meat without restriction	12-day weight record. Validated against 7-day food record.	Fasting blood samples. Measured: Hb, Hct and SF	**Hb (g/L):** Veg = 130 ± 8 Omn = 134 ± 8 (NS) **SF (ng/L):** Veg = 25.0 ± 16.2 Omn = 45.5 ± 42.5 (*p* < 0.025) **Correlation between meat and SF in Omn:** *R* = 0.03, *p* = 0.88 (NS)	More vegetarians consumed iron supplements than omnivores. More omnivores donated blood regularly.
Beck *et al.* (2013) [32]	Female, 18–44 years	Chicken, turkey, duck and beef	Iron Food Frequency Questionnaire (FeFFQ), determine dietary patterns and practices.	Blood sample measured: Hb, SF and CRP.	**Meat and vegetable intake** (Q1 = lowest intake, Q5 = highest intake). **SF (ng/L):** Q1 = 29 Q5 = 48 (*p* < 0.001) **Hb (g/L):** Q1 = 129 Q5 = 133 (*p* < 0.001) **Sub-optimal iron status:** Q1 = 21% Q5 = 5% (*p* < 0.001)	Cross sectional design. Bias regarding subject selection. Non-validated data collection methods. Not just based on animal flesh intake, but also vegetables, rich in Vitamin C.
Beck *et al.* (2014) [31]	Female, 18–44 years	Meat (beef), Chicken (turkey, duck), prepared meat, fish, seafood	Iron Food Frequency Questionnaire (FeFFQ).	Blood sample measured: Hb, SF and CRP	**After adjusting for other variables:** Meat and vegetable dietary pattern (SD) × having children (OR = 0.17), (95% CI for OR = 0.08, 0.39), (*p* < 0.001). Meat and vegetable dietary pattern (SD) × no children (*p* > 0.05)	Small convenience sample of women. Possible selection bias. Cross sectional design means causality could not be determined.
Blanco-Rojo *et al.* (2014) [33]	Female, 18–35 years	Red meat = beef, lamb, veal and pork. White meat = chicken and turkey. Processed meat = cured and smoked meats, ham, bacon, sausages, chorizo	72-h detailed diet reported. Validated in assessing nutrient and food intakes.	Fasting blood sample, measured: Hct, MCV, Hb, sFe, SF, sTf, Tf saturation, Total IBC, sTfR	**3 clusters of Iron status (C1 = IDA, C2 = Mild ID, C3 = Normal iron status).** **Consumption of red meat:** C1 = 42.2 ± 48.4 g/day C2 = 35.5 ± 40.4 g/day C3 = 75.8 ± 66.0 g/day **red meat intake b/n C3 and C1** (*p* < 0.05)	72-h record, unlikely to reflect habitual intakes and is self-reported data.
Brussaard *et al.* (1997) [35]	Male (50% and Female, 20–79 years	Meat, meat products and fowl.	3-day food record	Measured: sFe, Total IBC, Tf saturation, SF, Hb, Hct, MCHC MCH, MCV	Spearman correlation coefficient: **SF and meat intake:** Men: *r* = 0.15 (*p* < 0.05) Women: NS **Hb and meat intake:** Men: *r* = 0.15 (*p* < 0.05) Women: NS	3-day record unlikely to reflect habitual intakes. Non-representative population.
Cade *et al.* (2005) [36]	Female, 35–69 years	Vegetarian = eat meat or fish <1/week Oily fish = eat oily fish 2–4/week and meat <1/week. Meat eaters = eat meat >1/week.	FFQ consisting of 217 foods, classified by frequency of consumption. Validated tool.	Measures: SF, sFe, unbound IBC and Hb.	**SF:** Daily red meat eaters had 36% higher SF than non-consumers (*p* < 0.001). Fish was not associated with better SF (*p* = 0.2).	SF should be measured with CRP, to identify if raised SF is due to infection.
Deriemaeker *et al.* (2011) [38]	Male (24%) and Female, >65 years	Ovo-lacto-vegetarian = dairy and eggs in the diet, but no meat. Non-vegetarians = include meat in their diet	Validated semi-quantitative 104 item FFQ. Collecting standard portion sizes and 9 possible food frequency categories.	Fasting blood samples, measured: Hb, RBC, sFe, Tf and SF following validated procedure.	**Hb (g/L):** Men Veg = 13.4 ± 0.9 Men Non-Veg = 13.6 ± 1.2 (NS) Female Veg = 12.8 ± 1.1 Female Non-Veg = 13.1 ± 1.6 (NS) **SF (ng/L):** Men Veg = 47 ± 26 Men Non-Veg = 161 ± 165 (NS) Female Veg = 70 ± 46 Female Non-Veg = 158 ± 180 (NS)	Comparing elderly of two different locations. Data collection tool has short coming typical for a European diet. Non-representative sample.
Doyle *et al.* (1999) [39]	Males (51%) and Female, >55 years	Meat, Poultry and fish	Interviewers weighted 1 main meals for 4 days. Participants or careers were asked to keep a descriptive record of food and drink consumed for 24-h	Fasting blood sample, collected measured: SF, Hb, MCV, Hct, MCH, Total IBC, sFe	**Positive relationship between Hb and intake of meat, poultry and fish:** Men: *p* < 0.001 Women: *p* = 0.002 **Positive relationship with meat and sFe:** Men: *p* < 0.01 Women: NS	Unlikely that participants who provided blood samples were representative of the population.
Fleming *et al.* (1998) [40] (2002) [41]	Male (40%) and Female, 67–93 years	Poultry, Meat, processed meat, fish	Validated, 126 item semi-quantitative FFQ.	Non-fasting blood sample, measured SF and CRP	Meat significant positive predictor of SF (*p* = 0.0015). Each serve (113.6–170 g) of meat/week was associated with 6% greater SF or 20% per 3 serves of meat/week. Poultry and fish were NS related to SF.	Blood samples were non-fasting. SF was only iron status indicator measured.
Galan *et al.* (1998) [42]	Female, 35–60 years	Meat (including poultry) and fish	24-h diet record, collected every 2 mo (6 per year) on computer software. Portion sizes validated.	Fasting blood samples, measure: Hb and SF	Positive correlation between SF and meat (*r* = 0.18, *p* < 0.001) Positive correlation between SF and meat and fish (*r* = 0.05, *p* < 0.02)	Raised SF levels due to infection was not investigated.
Haddad *et al.* (1999) [43]	Male (44%) and Female, 20–60 years	Vegans = no animal products. Non-vegetarians = no restriction (consumed on average 176.4 g/day meat).	24-h recall was completed by trained interviewers	Fasting blood samples, measured: SF	**SF in males (ng/L):** Vegans = 72 Non-Veg = 141 (*p* < 0.05) **Compromised Iron stores (SF < 12 ng/L)** Vegan = 4/15 Non-Veg = 2/10	Different intakes of iron absorption inhibitors and enhancers.
Harman *et al.* (1998) [44]	Male (49%) and Female, 20–60 years	Non-Vegetarian = no restriction Some may exclude red meat. Vegetarians = no meat products, fish or poultry.	Subjects kept 7-day diet records. Kept pictures of serving sizes to aid with portion estimation.	Fasting blood samples, measured: Hb and SF	**Hb (g/L):** Male Non-Veg = 142 Male Veg = 150.5 (NS) Female Non-Veg = 129.3 Female Veg = 123.6 (NS) **SF (ng/L):** Male Non-Veg = 148.0 Male Veg = 79.8 (NS) Female Non-Veg = 59.6 Female Veg= 50.4 (NS)	Non-representative sample. Raised SF due to infection is not clear.
Harvey *et al.* (2005) [45]	Female, 18–45 years	RM group = red meat >5/week, plus poultry and fish P/F group = poultry and fish >5/week and 90 g pork or ham/week. V = no meat or fish in past year	7-day duplicate collection (participants collect exact duplicates of all foods and drinks consumed in each 24-h period)	3 x fasting blood samples, measured: SF, CRP, Tf saturation, Total IBC, TfR, Hb and MVC	**Hb (g/L):** RM = 134 P/F = 137 V = 135 (NS) **Median SF (ng/L):** RM= 6.8 (*p* < 0.01 lower than P/F) P/F = 17.5 V = 11.1 (NS from other groups) **TfR (mg/L):** RM = 3.19 P/F = 2.63 (*p* = 0.002, lower than V group) V = 3.92	Median SF was reported rather than mean SF concentrations.
Heath *et al.* (2001) [46]	Female, 18–40 years	Meat, fish and poultry	Iron food frequency questionnaire. 24-h recalls measured macronutrient intakes	Fasting blood sample, measure: SF, Hb and CRP	**With MID consumed:** 86 g/day **Without MID consumed:** 111 g/day (*p* = 0.002). **% of groups avoiding red meat:** With MID: 29.9% Without MID: 19.9% (*p* = 0.05)	Cross-sectional design. Non-representative population. 24-h recalls non-representative of habitual intakes.
Helman *et al.* (1987) [47]	Male (42%) and Female, >18 years	Vegetarian = no meat. Omnivores = include flesh foods in the diet.	Self-identified dietary habits.	Blood samples, measure: SF	**SF (ng/L):** Vegetarian = 45 Omnivore = 70 (*p* < 0.05)	Cross sectional design. Non-representative sample. Methods of dietary data collection and iron status not valid.
Houston *et al.* (1997) [48]	Female, 19–26 years	Beef, Pork or Lamb. Frequent intake >3 serves red meat/week. Infrequent intake 0.75–1.25 serves /week.	National cancer institute—health habits and history questionnaire.	Fasting blood sample, measured: SF, Hb, Hct, Total IBC and Tf saturation	Frequent red meat eaters had significantly greater Hb and hct levels compared to abstainers (*p* < 0.05) SF was greatest in red meat abstainers (NS).	Iron supplement users were not controlled for between meat eating groups. Unclear if diet data collection was valid or if inflection was considered with SF levels.
Hua *et al.* (2001) [49]	Male (25%) and Female, 35–45 years	Lacto-ovo-vegetarian = no meat for >5 years Meat eaters = eat meat at least once daily.	Self-reported diet habits	Fasting blood samples measure: SF	**SF (ng/L):** Veg = 35 Meat eaters = 72 (*p* = 0.0012)	SF was the only measure of iron status, and identification of diet group was non-valid.
Kato *et al.* (2000) [52]	Female, 34–65 years	Meat (red unprocessed and processed, white meat (poultry)) and fish.	Validated, self-administered semi-quantitative diet questionnaire.	Non-fasting blood sample, measure: Mean SF, sFe and IBC	**SF (ng/L) based on meat intake:** consume <42.4 g/day = 66.8 consume 42.4–66.9 g/day = 68.7 consume 66.9–104.5 g/day = 85.8 consume >104.5 g/day = 88.2 (*p* < 0.01) Subjects consuming the highest quartile for meat had 30% greater SF than those in the lowest quartile for meat consumption.	CRP was not measured and blood sample was non-fasting.
Kim *et al.* (2012) [53]	Female, 47–85 years	Vegetarian = no animal flesh food included in diet. Non-Vegetarian = include animal flesh food in diet	24-h recall for 3 days; used food models, dishes, glasses and spoons of different sizes for portion size estimation	Fasting blood sample, measure: SF levels	**SF (ng/L):** Vegetarian: 33.08 Non-Vegetarian: 44.33 (*p* < 0.01) Vegetarians consumed significantly more (*p* < 0.001) total and plant Fe than non-vegetarians, and consumed significantly less animal Fe (*p* < 0.001)	SF was the only Fe status indicator, thus SF may be influenced by a variety of factors other than meat intake.
Leonard *et al.* (2014) [54]	Female, 18–35 years	Beef, chicken, lamb, veal, sausages, fish, bacon, ham and salami.	Validated FFQ, including 74 items. Semi-quantitative and self-administered.	Blood samples, measured SF, Hb and sTfR	**Positive correlation** SF and frequency of flesh consumption (*r* = 0.31, *p* = 0.01), SF and all meat consumption (*r* = 0.27, *p* = 0.01).	Cross sectional design. Non-representative sample. Dietary intakes based on self-reported data. Unclear if blood samples are fasting.
Levin *et al.* (1986) [55]	Male (55%) and Female, >18 years	Ovo-lacto-vegetarian and omnivore (Not otherwise defined)	Detailed food frequency, cross checked with 24-h recall.	Blood tests measure: sFe and TIBC	**sFe (ng/L):** NS difference between diet groups **TIBC (ng/L):** Men Omn = 393.3 Men Veg = 351.6 (*p* < 0.001) Female Omn = 409.01 Female Veg = 361.8 (*p* < 0.001)	Males and females are unevenly distributed between groups. Non-standard iron status indices.
Ley *et al.* (2014) [56]	Female, 30–50 years	Unprocessed meat = beef, lamb, pork. Processed meat = bacon, hot dog, salami. Poultry = chicken and turkey. Fish = dark and light fish and canned tuna	Validated semi-quantitative 131-item food frequency questionnaire.	Blood sample, measured: CRP, SF and TfR	**Subjects split into quartiles based on meat consumption.** Q1 = 36.1 g/day, Q2 = 46.1 g/day, Q3 = 46.9 g/day, Q4 = 51.8 g/day (*p* = 0.0002) If 1 serve of red meat was substituted for alternative protein, this lead to a reduction in SF, except with poultry (β = 0.054 ± 0.260, *p* = 0.39).	Higher CRP and SF were associated with higher red meat intakes. Biomarkers were measured from sub-studies.
Locong *et al.* (1986) [57]	Male (35%) and Female, >18 years	Vegetarian = meat, poultry or fish 1/mo. Non-vegetarians = flesh foods more than 1/mo.	3-day food diaries comprising of 2 weeks days and 1 weekend day.	Fasting blood samples measure: SF	**SF (ng/L):** Vegetarians: 55 ± 40 Non-Vegetarians: 74 ± 54 (NS)	Self-reported diet data, not all blood samples were fasting. Only measured SF.
Milman *et al.* (2004) [59]	Male (48%) and Female, >80 years	Meat (not otherwise defined)	Diet History: 3-day food record and food frequency checklist	Non-fasting blood samples, measured:Hb, SF and CRP	**Positive correlation between meat intake and SF** (*r* = 0.16, *p* = 0.013). Men: *r* = 0.16, *p* =0.08 Women: *r* = −0.01, *p* = 0.95	Non-representative sample. Meat was undefined. Non-fasting blood samples.
Patterson *et al.* (2001) [61]	Female, >18 years	Meat, fish and poultry	7-day weighted food record	Blood test measured: SF, Hb, sFe and Total IBC	NS different meat intake found between ID and Fe replete women. ID women consumed = 90 ± 56.5 g Fe replete women consumed = 104.7 ± 45.9 g	Non-representative sample. Small sample size.
Rigas *et al.* (2014) [63]	Male (54%) and Female, 18–67 years	Meat, fish and poultry	Questionnaire, collected average weekly intake for food and beverages	Measured: SF and Hb from blood sample	Meat intake positively associated with iron stores, while consumption of fish was only positive for men. Meat consumption reduced risk of ID in men (*p* = 0.003) and premenopausal women (*p* = 0.004)	Non-representative sample. Unclear if blood sample was fasting or if inflammation was considered with SF levels.
Schuepbach *et al.* (2011) [65]	Male (21%) and Female, 24–55 years	Red meat (not otherwise defined)	Participants asked to provided estimated amount of red meat consumption/week in grams	Blood sample, measured: CRP, Hb, MCV, SF. sFe, sTf and sTfR	Red meat intake was low among the 15 subjects with SF < 15 µg/L. The amount of red meat consumed and SF values correlated significantly, but weakly (*r* = 0.025).	Small, non-representative sample. Only reported on red meat. Diet collection method was non-validated, with high risk of bias. Unclear if blood samples were fasting.
Seaverson *et al.* (2007) [66]	Male (41%) and female, 51–91 years	White and red meat (Not otherwise defined)	FFQ, with 3D models to estimated portion sizes.	Fasting blood sample, measured: MCV, Hb, Hct, SF and CRP	Haem iron from red meat, positively associated with SF (*r* = 0.46, *p* = 0.02). haem iron from white meat was negatively associated with SF (*r* = −0.28, *p* = 0.12) Each additional serve of red meat was associated with 5% greater SF levels	Cross sectional study. Meat not clearly defined.
Snyder *et al.* (1989) [67]	Female, >18 years	Red meat (RM) group = eat red meat (>100 g/week) Modified Vegetarian (MV) group = eat eggs, milk, fish, poultry and <100 g/week or no red meat.	Questionnaire, collects dietary habits to identify if participants belonged to either RM or MV group.	Fasting blood samples, measured: Hb, Hct, sFe, TIBC and SF.	**Hb (g/L):** MV = 13.5 ± 0.2 RM = 13.1 ± 0.3 (NS) **SF (ng/L):** MV = 7.4 ± 1.4 RM = 19.8 ± 4.2 (*p* < 0.05) **Number of subjects with SF < 12 (ng/L):** MV = 8/9 RM = 2/9 (*p* < 0.05)	Small sample size. Meat consumption data was collected using non-validated methods.
Thomson *et al.* (2011) [68]	Female, 50–79 years	Red meat (not otherwise defined)	Validated, FFQ (baseline and at year 3), portion sizes and frequency was collected	Fasting blood samples, measure: Hb	Women without anaemia had higher red meat intake than those with (*p* < 0.01). A greater consumption of red meat was marginally associated with lower risk of persistent anaemia.	Self-reported dietary data. Anaemia cut-off points based on Hb. Non-representative population.
Vaquero *et al.* (2004) [69]	Male (41%) and Female, >70 years	Meat, meat products and fish	7-day weighed food record.	Fasting blood samples, measured: Hct, Hb, MCV, MCHC and SF	Significant positive correlation between SF and meat intake *r* = 0.403, *p* = 0.01	Non-representative sample. Cross sectional design.
Wilson *et al.* (1999) [71]	Male, 20–50 years	Vegetarian = never eat red meat or chicken, but eat fish no more than 1/week. Vegan = no meats, eggs or dairy. Omnivore = no meat restrictions	12-day semi-qualitative food record on either 12 consecutive days for from 3 sequences of 4 consecutive days, but included 3-4 days.	Fasting venous samples, measured: SF and Hb	**SF (ng/L):** Omnivore: 121 ± 73 Ovo-lacto-vegetarian: 64 ± 47 (*p* < 0.001, different from omnivore) Vegan: 65 ± 50 (*p* < 0.05, different from omnivore) **Hb (g/L):** Omnivore: 173 ± 19 Ovo-lacto-vegetarian: 140 ± 40 (*p* < 0.001, different from omnivore) Vegan: 158 ± 28 (*p* < 0.05, different from omnivore)	Diet history is self-reported data. Cross-sectional cannot determine causality.CRP was not measured.Non-representative sample.
Wittenbecher *et al.* (2015) [72]	Male (39%) and Female, 35–64 years	Red meat: un-processed (beef, veal, pork and lamb) and processed meats (bacon, ham, sausages)	Validated semi quantitative FFQ	Blood sample, measured SF	**SF was significantly associated with total red meat consumption in both sexes:** Women (*p* < 0.001) Men (*p* = 0.002)	Dietary information based on habitual intakes over the past year, and metabolites were measured at a single point in time. SF was the only biomarker measured relating to iron status.
Worthington-Roberts *et al.* (1988) [73]	Female, 20–50 years	RM group = Red meat >5 times/week, PF group = Poultry and fish as major protein. Red meat <1/week. V group = no meat.	24-h recalls, as well as 3-day records (including 1 weekend day).	Fasting blood samples, measured: SF, sFe and Total IBC	**SF (ng/mL):** RM = 30 PF = 15 V = 20 (*p* = 0.01) Hb (Gm/dl): RM = 14 PF = 13.4 V = 13.5 (*p* = 0.026) **TIBC (ng/L):** RM = 350 PF = 360 V = 355 (*p* = 0.04)	Cross-sectional and small sample size.
Yokoi *et al.* (1994) [74]	Female, 19–40 years	Red meat, poultry, fish and shell-fish	FFQ	Blood samples, measured: SF	Correlation coefficient between SF and red meat intake *r* = 0.331, *p* = 0.04. Correlation between SF and other flesh (poultry, fish and seafood) were less than 0.025. Multiple regression between red meat and SF had positive weights *r* = 0.596, *p* = 0.0002	Infection was not accounted for when measuring SF, and was the only iron status indicator used.
Yokoi *et al.* (2007) [75]	Female, 19–39 years	Beef (not otherwise defined)	Self-administered FFQ	Fasting blood samples, measured: Hb, SF and sFe	Beef consumption correlated positively with SF (correlation coefficient = 0.645, *p* < 0.001)	Self-reported diet data, unclear if infection was considered with SF

SF = Serum Ferritin; Hb = Haemoglobin; CRP = C-Reactive Protein; Hct = Haemotocrit; NS = Not Significant; sTfR = serum Transferrin Receptor; sFe = serum iron; IBC = Iron binding capacity; FFQ = Food Frequency Questionnaire; RBC = Red Blood Cells; Tf = Transferrin; MCV = Mean Corpuscular Volume; MCHC = Mean Corpuscular Haemoglobin Concentration; MCH = Mean Corpuscular Haemoglobin; MID = Mild Iron Deficiency.

## 4. Discussion

Iron deficiency remains the most widespread nutrient deficiency of the developed world [1,2]. Dietary modification to include greater quantities of animal flesh foods may be a relatively simple intervention for improving iron status or preventing ID. This is especially important for women of childbearing age, who tend to experience the highest rates of iron deficiency, yet often consume the lowest intakes of flesh foods [59,63,65,69]. To the authors’ knowledge, this is the first systematic review to examine systematically whether a higher consumption of animal flesh foods results in better iron status for adults within developed countries.

Overall, the results of the included studies appear highly conflicting, a likely outcome of the broad variety of study participants and study designs included in the review. Women of childbearing age featured heavily across the literature as they are at great risk of developing ID due to their high iron demands secondary to menstruation and pregnancy [4]. However, male adults, elderly populations and postmenopausal women have also featured in some of the included studies.

Seven studies rated positive in quality, to which three were experimental [14,34,60] and four were observational studies [46,62,66,74]. Six of these studies focused on female populations, specifically women of childbearing age [14,34,46,60,62,74]. Of these studies, animal flesh intake appeared to have a positive effect on the iron status of women of childbearing age [14,34,46,60,74]. However, while Blanton [34] found a moderate consumption of beef resulted in significantly higher SF and Hb concentrations at 16-weeks, the number of women who experienced a SF response during the intervention was not different between beef and non-beef lunch groups. Further indicating that the iron status of premenopausal women was more strongly predicted by baseline iron status, such that women with lower baseline iron status experienced a greater degree of iron status improvement during the intervention regardless of beef consumption. This finding is not surprising considering multiple studies have indicated that the primary determinant for iron absorption is the systematic need for iron [4,76,77] and thus may account for why other studies [29,45,51,61] found no relationship between animal flesh intake and iron status in women of childbearing age.

Noakes *et al.* [60] and Cheng *et al.* [37] placed women on either a high protein diet rich in animal flesh or a high carbohydrate diet low in animal flesh, designed with weight loss as the primary outcome. In both studies, the high-protein-high-meat diet group experienced the greatest amount of weight loss, and additionally a better iron status by the end of the intervention. This is an important finding, as it has been found that women who habitually diet for weight loss are at an increased risk of developing poor iron status [48]. Thus, the consumption of meat as part of a weight loss intervention in women may have a protective effect on iron status. In addition, exercise has been linked to depleted iron stores, as iron losses are increased through sweat and in extreme cases gastrointestinal bleeding and haematuria [4]. In a study conducted by Lyle *et al.* [58], a meat based diet was shown to protect the iron status of exercising women of childbearing age. Further Snyder *et al.* [67] found that female distance runners consuming low levels of animal flesh and no red meat had significantly lower SF concentrations than women who consumed red meat, indicating that a higher consumption of animal flesh foods, specifically red meat may have a protective effect on the iron status of exercising females.

Conflicting results have been found among the observational studies in this population sub-group. While Heath *et al.* [46] and Yokoi *et al.* [74] found that women consuming greater quantities of animal flesh and red meat had better iron status and reduced risk of ID, Pynaert *et al.* [62] found there was no association between all flesh food intake and iron status in this population, a finding that was repeated in a large Japanese sample (*n* = 1019) [29]. However, it is also important to note that significant non-dietary factors such as the frequency and intensity of menstrual blood flow and the use of hormonal contraceptives are also associated with ID risk in this population [29,61]. Thus, the factors influencing the iron status of premenopausal women is wrought with complexity and significant confounders, likely influencing the outcomes of these observational studies. Further, it is important that future studies in this population appropriately adjust for these factors to better understand the role animal flesh intake has in relation to menstrual blood losses, use of hormonal contraceptives and number of pregnancies. However, overall the consumption of animal flesh foods in women of childbearing age appears to have a protective effect on iron status, with most of the positive and neutral quality studies indicating this [32,33,54,75]. Additionally, these findings are consistent with those reported in a recent systematic review which concluded, for young women living within industrialised countries, the consumption of meat is associated with increased iron status [76].

Of the large observational studies that included pre and post-menopausal women [36,56], there appeared to be a positive association between total flesh intake and iron status, in which the consumption of red meat was most significantly correlated with iron status. Some studies have shown greater iron status indices in postmenopausal women than premenopausal women [42,63], due to ceased menstrual blood losses and lower body iron demands. In a large prospective cohort study [68] (*n* = 72,833), conducted on a postmenopausal female population, it was found that a greater red meat consumption was reported in women without ID anaemia, indicating that the consumption of red meat may also lower the risk of ID in this population.

Post-menopause, women’s iron requirements should be reduced, and studies have indicated that iron status differences between men and women are lessened as a result [78]. A cross-sectional study, which rated positive in quality, focused on an elderly population of both females and males, and found that red meat was positively associated with SF concentrations, but that white meat was negatively correlated with SF [66]. This finding has been reported by other large observational studies focusing on an older population group, indicating that meat, and in particular red meat intake, was positively associated with iron status [39,40,41]. In the context of dietary sources of bioavailable iron, these results are to be expected given that red meat is a relatively rich source of high-bioavailable haem iron, providing approximately 1.1–1.3 mg/100 g, as opposed to other sources of animal flesh such as chicken and fish providing around 0.1–0.9 mg/100 g [4,15,79]. Red meat also contains greater quantities of low-bioavailable non-haem iron, and the presence of the non-haem enhancer Meat Protein Factor (MPF) may improve the amount of this iron absorbed [4,79]. Additionally, there have been multiple reports that the type of dietary iron consumed is a better determinant of iron status than total iron dietary intake [4,76].

Mixed results were found across the studies comparing meat containing diets and vegetarian diets, an outcome that should be expected considering the vastly different dietary factors in addition to animal flesh intake that may influence the iron absorption and iron status of subjects. A study conducted by Tetens *et al.* [14] was the only study comparing the two diet types that rated positive in quality. The study was a 20 weeks RCT, conducted with women of childbearing age comparing the effect of a meat containing diet to a vegetarian diet [14]. At the end of the intervention, women consuming the meat based diet showed increased SF values, compared with the vegetarian diet group which experienced a significant reduction in SF and Hb [14]. This finding is particularly important given that the vegetarian based diet group on average consumed more total iron (12.3 ± 0.3 mg/day) compared with the meat base diet group (11.0 ± 0.5) during the intervention.

While the observational studies comparing the iron status of subjects consuming a vegetarian diet with an omnivorous diet were of either neutral or negative quality, most studied small populations, thus producing a very low level of evidence. However, the majority of these studies reported greater SF and iron status indices in subjects consuming an omnivorous diet compared with a vegetarian diet [28,30,43,47,49,53,64,70,71]. This further indicated that higher consumption and inclusion of animal flesh in the diet may be associated with a greater iron status for adults within developed countries.

Iron absorption is known to be influenced by the presence of various dietary enhancing and inhibiting factors, and there is also good evidence to suggest that dietary behaviors such as the combination in which foods and beverages are consumed throughout the day may influence iron absorption and, further, the iron status of adults [4,76]. It is interesting to note that only Beck and colleagues [31,32] investigated iron status in relation to dietary patterns. Beck *et al.* [32] found subjects with the highest intakes of meat and vegetables had significantly greater SF and Hb values and reduced risk of ID than those consuming the least meat and vegetables. Further a high milk and yoghurt or coffee and tea dietary pattern was associated with increased risk of poor iron status [32]. Yet participants with sufficient iron stores were more likely to consume milk or milk products between meals [32].

A combination of dietary approaches such as increasing intake of haem iron and iron enhancers, reducing intake of inhibitors and considering the timing of iron enhancers and inhibitors with meals have been recommended [4,76]. More studies examining iron status and meat intake in the context of the whole diet need to be conducted to gain a better understanding of this area. This may be of particular relevance given the recent development of technology based dietary intake tools. Developments such as electronic diaries based on audio recordings and photos of foods consumed, may provide a more accurate means of assessing habitual food intakes and behaviors, than traditional methods [80].

### Limitations of Included Studies

The presence of confounding factors across the studied populations such as gender, age and lifestyle may have a very prominent effect on the variation in results found in this review. Additionally, factors known to influence iron status such as use of iron supplements, blood donation, exercise, ethnicity and Body Mass Index (BMI) differ across the participants of all 49 included studies, and may play a role in the direction of study outcomes. It is also worth noting that in women of childbearing age, other confounding factors such as menstrual blood losses, use of contraceptive medication and the number of births were often not considered and may be a major limitation of the studies focusing on this population group.

The studies included in this review were primarily of cross-sectional design, providing a low level of evidence to which causality cannot be applied. An additional limitation common across many of the included studies’ methodologies was the use of small and non-representative samples. It was common for study sampling to rely on volunteers of non-representative populations such as university students and staff or health professionals. Further, it was common across studies to use self-reported dietary data collection techniques, or non-validated dietary data collection tools which may contribute to participant bias. Three day diet records were commonly used as a measure of dietary patterns, however, in reality this is unlikely to reflect habitual intake of flesh foods, particularly any one type of flesh food, such as red meat, poultry or fish.

Experimental studies, while they tended to attract a higher quality rating, were limited by the requirement of participants to adopt a specific diet. As a result, these studies were not able to blind participants to their designated diet group. They were also unable to strictly control dietary intake of free living adults, thus introducing a potential source of bias.

Overall, only seven out of 49 studies were assigned a positive quality rating based on criteria outlining study generalizability, bias and validity. Thirty eight studies rated neutral and four negative, indicating that the overall generalizability of these study outcomes is low and/or study bias is high and is an underlying limitation of this review.

Studies included in this review were not just widely varied in population and study design, but also in the independent variable of interest. Some measured the total amount of animal flesh consumed; some separated total animal flesh into red and white flesh, while others measured only red flesh or beef consumption. Further, the question of whether a higher consumption of all animal flesh food is associated with a better iron status among adults within developed countries is not completely elucidated by these studies. In addition, the definition of a vegetarian diet varied between studies, with some studies allowing “vegetarians” to consume meat occasionally, while others were strictly no meat. Others may not have included egg and/or dairy foods, as the definition of a vegetarian was not always provided.

Measurement techniques of the dependent variable were diverse and the use of standardized techniques was not always clearly defined. Measurements based on non-fasting blood samples need to be considered with caution, as serum iron levels can be directly influenced by recent dietary intakes. Not all SF measurements were considered with C-Reactive Protein (CRP) levels; therefore, it was unclear if SF levels were raised in reflection of greater iron stores or in response to infection and inflammation [4].

Intakes of animal flesh and the effect on iron status was analysed very differently across studies, some taking into account confounders to varied degrees, thus making the true direction and strength of effect difficult to determine.

## 5. Conclusions

This review aimed to investigate whether a higher consumption of animal flesh foods results in better iron status among adults (>18 years) living within developed countries.

As expected, a large proportion of studies conducted in this area were of women of childbearing age, as they are the sub-group at greatest risk of developing poor iron status amongst adults. Given the heterogeneity of studies even in this sub-group, it may be more appropriate in future reviews to consider investigating the effect of animal flesh foods on iron status in specific population sub-groups such as; women of childbearing age, post-menopausal women, men, and elderly adults.

Many cross-sectional studies investigating flesh consumption and iron status have been conducted; but more good quality longitudinal and experimental studies are needed in order to determine a clear effect. These studies should investigate flesh food intake amount and frequency, as well as associated dietary patterns that effect iron absorption, all of which are important in determining iron status. Additionally, other factors that may influence iron status such as menstrual blood loss, contraception, exercise and dieting patterns should also be measured and considered in order to fully understand the role of flesh food intake in determining iron status.

While results across the 49 included studies appear highly conflicting, over all there appears to be a positive association between the intake of animal flesh foods and the iron status of adults within developed countries based upon the positive quality and larger studies conducted. Whether there is an optimum quantity or frequency of flesh intake required to maintain or achieve a healthy iron status in adults remains unclear.

## Supplementary Materials

The following are available online at http://www.mdpi.com/2072-6643/8/2/89/s1, Table S1: Database search terms, Table S2: Study characteristics of included studies, Table S3: Results of study quality based on the Academy of Nutrition and Dietetics Quality Criteria.
