# Supplementary material for: Is Higher Consumption of Animal Flesh Foods Associated with Better Iron Status among Adults in Developed Countries? A Systematic Review
Source: Nutrients. 2016 Feb 16;8(2):89. doi: 10.3390/nu8020089 (PMC4772052; doi:10.3390/nu8020089)
Supplement: Supplementary file 1 [file nutrients-08-00089-s001.docx]

Supplementary Materials: Is Higher Consumption
of Animal Flesh Foods Associated with Better Iron Status among Adults in Developed Countries:
A Systematic Review

Jacklyn Jackson, Rebecca Williams, Mark McEvoy, Lesley MacDonald-Wicks
and Amanda Patterson

**Table S1.** Database search terms.

| 1. | Meat/or poultry/or seafood/or shellfish/ |
| --- | --- |
| 2. | Heam iron.mp. |
| 3. | Red meat.mp. |
| 4. | ((beef or poultry or chicken or muscle meat or fish or pork or lamb or seafood * or carcass meat) adj5 (eat * or food * or intake or consum * or diet *)).mp. (mp = title, abstract, original title, name of substance word, subject heading word, keyword heading word, protocol supplementary concept word, rare disease supplementary concept word, unique identifier) |
| 5. | Non vegetarian *.mp. |
| 6. | Omnivore.mp. |
| 7. | Anemia, iron-deficiency/ |
| 8. | Iron deficiency.mp. |
| 9. | Serum ferritin.mp. |
| 10. | Receptors, Transferrin/or soluble ferritin receptor.mp. |
| 11. | Hemoglobins/or haemoglobin.mp. |
| 12. | Iron stores.mp. |
| 13. | Iron status.mp. |
| 14. | Iron/ |
| 15. | Anemia/ |
| 16. | 8 not 15 |
| 17. | 1 or 2 or 3 or 4 or 5 or 6 |
| 18. | 7 or 8 or 9 or 10 or 11 or 12 or 13 or 14 or 15 or 16 |
| 19. | 17 and 18 |
| 20. | Limit 19 to humans |
| 21. | Limit 20 to English language |
| 22. | Limit 21 to “all adult (19 plus years)” |

Please note * is a truncation used in database searches to ensure that all possible variations of a search term have been retrieved.

**Table S2.** Study characteristics of included studies.

| **Author, Country** | **Quality Rating** | **Study Design** | **Participants (*n*, Gender, Age)** |
| --- | --- | --- | --- |
| Alexander *et al.* (1994)[28], New Zealand | Ø | Cross sectional: V and Omn iron status comparison | 105, male (27%) and female (73%), >18 years |
| Askakura *et al.* (2009) [29], Japan | Ø | Cross sectional: meat or fish intakes compared to prevalence of ID | 1019, female, 18–25 years |
| Ball *et al.* (1999) [30], Australia | Ø | Cross sectional: V and Omn iron status comparison | 74, females, 18–45 years |
| Beck *et al.* (2013)[32],  New Zealand | Ø | Cross sectional: iron status compared to dietary intakes of meat and vegetables | 375, females, 18–44 years |
| Beck *et al.* (2014) [31],  New Zealand | Ø | Cross sectional: iron status compared to dietary intakes of meat and vegetables | 375, Females 18–44 years |
| Blanco-Rojo *et al.* (2014) [33], Spain | Ø | Cross sectional: iron status compared to dietary meat intake | 142, females, 18–35 years |
| Blanton (2014) [34],  United States | + | 16-wk Randomised controlled trial: Beef lunch group *vs.* non beef lunch group | 56 randomised (77% retention rate), females, 18–30 years |
| Brussaard *et al.* (1997) [35], Netherlands | - | Cross sectional: meat intake compared to SF concentrations | 444, males (50%) and female (50%), 20–79 years |
| Cade *et al.* (2005) [36],  United Kingdom | Ø | Prospective cohort: meat intake compared to SF concentrations | 6779, female, 35–69 years |
| Cheng *et al.* (2013) [37], Australia | Ø | 12-mo Random controlled trial: High protein-high meat diet *vs.* High carbohydrate—low meat diet | 71 randomised (51% retention rate), Female,  18–25 years |
| Deriemaeker *et al.* (2011) [38], Netherlands | Ø | Cross sectional: V and Omn iron status comparison | 59, male (24%) and female (76%), >65 years |
| Doyle *et al.* (1999) [39],  United Kingdom | Ø | Cross sectional: iron status compared to meat, fish and poultry intake | 1268, male (51%) and female (49%), >65 years |
| Fleming *et al.* (1998) [40], (2002) [41], United States | Ø | Cross sectional: iron status compared to meat, processed meat, poultry and fish intake | 634, male (40%) and female (60%), 67–93 years |

**Table S2.** *Cont.*

| **Author, Country** | **Quality Rating** | **Study Design** | **Participants (*n*, Gender, Age)** |
| --- | --- | --- | --- |
| Galan *et al.* (1998) [42], France | Ø | Prospective cohort: iron status compared with meat and fish intake | 3111, female, 35–60 years |
| Haddad *et al.* (1999) [43], United States | Ø | Cross sectional: V and Omn iron status comparison | 45, male (44%) and female (56%), 20–60 years |
| Harman *et al.* (1998) [44], New Zealand | Ø | Cross sectional: V and Omn iron status comparison | 47, male (49%) and female (51%), 20–60 years |
| Harvey *et al.* (2005) [45], United Kingdom | Ø | Cross sectional: red meat eater *vs.* poultry and fish eater and V diet comparison | 90, female, 18–45 years |
| Heath *et al.* (2001) [46],  New Zealand | + | Cross sectional: iron status compared to meat, fish and poultry intake | 384, female, 18–40 years |
| Helman *et al.* (1987) [47], Australia | - | Cross sectional: V and Omn iron status comparison | 173, male (42%) and female (58%), >18 years |
| Houston *et al.* (1997) [48], United States | Ø | Cross sectional: red meat intake compared to iron status | 80, female, 19–26 years |
| Hua *et al.* (2001) [49],  United States | Ø | Cross sectional: V and Omn iron status comparison | 60, male (25%) and female (75%), 35–45 years |
| Hunt *et al.* (1995) [50],  United States | Ø | 7 wk Randomised Controlled trial: High meat diet *vs.* low meat diet | 14 randomised (retention rate N/A), Female,  51–70 years |
| Hunt *et al.* (1999) [51],  United States | Ø | 8 wk Randomised cross over trial: V *vs.* Omn diet | 21 randomised (100% retention rate), female  20–42 years |
| Kato *et al.* (2000) [52],  United States | Ø | Cross sectional: meat intake compared to iron status | 487, female, 34–65 years |
| Kim *et al.* (2012) [53],  South Korea | Ø | Cross sectional: V and Omn iron status comparison | 107, female, 47–85 years |
| Leonard *et al.* (2014) [54], Australia | Ø | Cross sectional: intake of animal flesh compared to iron status | 107, female, 18–35 years |
| Levin *et al.* (1986) [55], Israel | - | Cross sectional: V and Omn iron status comparison | 205, male (55%) and female (45%), >18 years |
| Ley *et al.* (2014) [56],  United States | Ø | Cross sectional: red meat and processed meat intake compared to SF values | 3690, female, 30–50 years |

**Table S2.** *Cont.*

| **Author, Country** | **Quality Rating** | **Study Design** | **Participants (*n*, Gender, Age)** |
| --- | --- | --- | --- |
| Locong *et al.* (1986) [57], Canada | - | Cross sectional: V and Omn iron status comparison | 68, male (35%) and female (65%), >18 years |
| Lyle *et al.* (1992) [58], United States | Ø | 12 wk Randomised controlled trial: Exercise intervention, with a meat based diet, placebo or control | 83 (72% retention rate), female, >18 years |
| Milman *et al.* (2004) [59], Denmark | Ø | Cross sectional: meat intake compared to iron status | 362, male (48%) and female (52%),>80 years |
| Noakes *et al.* (2005) [60], Australia | + | 12 wk Randomised Controlled Trial: High-protein-high meat diet *vs.* High Carbohydrate-low-meat diet | 119 randomised (84% retention rate), Females, 20–65 years |
| Patterson *et al.* (2001) [61], Australia | Ø | Cross sectional: meat intake compared to iron status | 76, female, >18 years |
| Pynaert *et al.* (2007) [62], Belgium | + | Prospective cohort: V and Omn iron status comparison | 788, female, 18–39 years |
| Rigas *et al.* (2014) [63], Denmark | Ø | Prospective cohort: meat intake compared to iron status | 14,737, male (54%) and female (46%),  18–67 years |
| Reddy *et al.* (1990) [64], United Kingdom | Ø | Prospective cohort: V and Omn iron status comparison | 61, female20–40 years |
| Schuepbach *et al.* (2011) [65], Switzerland | Ø | Cross sectional: red meat consumption compared to iron status | 60, male (21%) and female (79%), 24–55 years |
| Seaverson *et al.* (2007) [66], United States | + | Cross sectional: meat intake compared to iron status | 604, male (41%) and female (59%), 51–91 years |
| Snyder *et al.* (1989) [67],  United States | Ø | Cross sectional: red meat intake compared to the iron status of modified vegetarian diets in long distance runners | 18, female, >18 years |
| Tetens *et al.* (2007) [14], Denmark | + | 20 wk Randomised controlled trial: meat based diet *vs.* vegetarian diet | 61, Female, 19–39 years |
| Thomson *et al.* (2011) [68], United States | Ø | Prospective cohort: red meat intake compared to iron status | 72,833, female, 50–79 years |
| Vaquero *et al.* (2004) [69], Spain | Ø | Cross sectional: meat intake compared to iron status | 110, male (41%) and female (59%), >70 years |

**Table S2.** *Cont.*

| **Author, Country** | **Quality Rating** | **Study Design** | **Participants (*n*, Gender, Age)** |
| --- | --- | --- | --- |
| Wells *et al.* (2003) [70], United States | Ø | 12 wk Randomised controlled trial: beef containing diet *vs.* vegetarian diet, during resistive training intervention | 26 randomised (retention rate 85%), male,  59–78 years |
| Wilson *et al.* (1999) [71], Australia | Ø | Cross sectional: V and Omn iron status comparison | 74, male, 20–50 years |
| Wittenbecher *et al.* (2015) [72], Germany | Ø | Prospective Cohort: serum ferritin concentrations compared to dietary intakes of red meat | 2047, Males (39%) and female (61%),  35–64 years |
| Worthington-Roberts *et al.* (1988) [73], United States | Ø | Cross sectional: meat intake compared to iron status | 52, female, 20–50 years |
| Yokoi *et al.* (1994) [74], United States | + | Cross sectional: meat intake compared to iron status | 19, female, 19–40 years |
| Yokoi *et al.* (2007) [75], United States | Ø | Cross sectional: beef intake compared to iron status | 33, female, 19–39 years |

V = Vegetarian; Omn = Omnivore; ID = Iron Deficiency; wk = Week; mo = Month; - = Negative quality rating; + = Positive quality rating; Ø = Neutral quality rating.

**Table S3.** Results of study quality based on the Academy of Nutrition and Dietetics Quality Criteria.

| **Author** | **Quality Rating** | **Relevance Q1** | **Relevance Q2** | **Relevance Q3** | **Relevance Q4** | **Validity Q1** | **Validity Q2** | **Validity Q3** | **Validity Q4** | **Validity Q5** | **Validity Q6** | **Validity Q7** | **Validity Q8** | **Validity Q9** | **Validity Q10** |
| --- | --- | --- | --- | --- | --- | --- | --- | --- | --- | --- | --- | --- | --- | --- | --- |
| Alexander *et al.* (1994) [28] | Ø | N/A | Yes | Yes | N/A | Yes | No | Unclear | Yes | Unclear | Yes | Unclear | Yes | No | Unclear |
| Askakura *et al.* ( 2009) [29] | Ø | N/A | Yes | Yes | N/A | Yes | No | Unclear | Yes | Unclear | Yes | Yes | Yes | Yes | Yes |
| Ball *et al.* (1999) [30]. | Ø | N/A | Yes | Yes | N/A | Yes | No | Yes | Yes | Unclear | Yes | Yes | Yes | No | Unclear |
| Beck *et al.* (2013) [32] | Ø | N/A | Yes | Yes | N/A | Yes | No | Yes | Yes | Unclear | Yes | Yes | Yes | Yes | Yes |
| Beck *et al.* (2014) [31] | Ø | N/A | Yes | Yes | N/A | Yes | No | Yes | Yes | Unclear | Yes | Yes | Yes | Yes | Yes |
| Blanco-Rojo  *et al.* (2014) [33] | Ø | N/A | Yes | Yes | N/A | Yes | Unclear | Yes | No | Unclear | Yes | Yes | Yes | No | Yes |

**Table S3.** *Cont.*

| **Author** | **Quality Rating** | **Relevance Q1** | **Relevance Q2** | **Relevance Q3** | **Relevance Q4** | **Validity Q1** | **Validity Q2** | **Validity Q3** | **Validity Q4** | **Validity Q5** | | **Validity Q6** | **Validity Q7** | **Validity Q8** | **Validity Q9** | **Validity Q10** |
| --- | --- | --- | --- | --- | --- | --- | --- | --- | --- | --- | --- | --- | --- | --- | --- | --- |
| Blanton (2014) [34] | + | Unclear | Yes | Yes | Yes | Yes | Yes | Yes | Yes | Yes | | Yes | Yes | Yes | Yes | Yes |
| Brussaard *et al.* (1997) [35] | - | N/A | Yes | Yes | N/A | Yes | No | Unclear | No | Unclear | | No | No | Yes | No | No |
| Cade *et al.* (2005) [36] | Ø | N/A | Yes | Yes | N/A | Yes | Yes | Unclear | Yes | Unclear | | Yes | Yes | Yes | Yes | Yes |
| Cheng *et al.* (2013) [37] | Ø | Yes | Yes | Yes | Yes | Yes | Yes | Yes | Yes | Yes | | Yes | Yes | Yes | Yes | Yes |
| Deriemaeker *et al.* (2011) [38] | Ø | N/A | Yes | Yes | N/A | Yes | Unclear | Unclear | No | Unclear | | Yes | Yes | Yes | Yes | Yes |
| Doyle *et al.* (1999) [39] | Ø | N/A | Yes | Yes | N/A | Yes | No | Yes | Yes | Unclear | | Yes | Yes | Yes | Yes | Yes |
| Fleming *et al.* (1998) [40], (2002) [41]. | Ø | N/A | Yes | Yes | N/A | Yes | Yes | N/A | Yes | Unclear | | Yes | Yes | Yes | No | No |
| Galan *et al.* (1998) [42]. | Ø | N/A | Yes | Yes | N/A | Yes | Unclear | Unclear | Yes | Yes | | Yes | Yes | Yes | No | Unclear |
| Haddard *et al.* (1999) [43] | Ø | N/A | Yes | Yes | N/A | Yes | No | Yes | No | Unclear | | Yes | Yes | Yes | Yes | Unclear |
| Harman *et al.* (1998) [44] | Ø | N/A | Yes | Yes | N/A | Yes | No | Yes | No | Unclear | | No | Unclear | Yes | No | No |
| Harvey *et al.* (2005) [45] | Ø | N/A | Yes | Yes | N/A | Yes | No | Yes | Yes | Unclear | | Yes | Yes | Yes | Yes | Unclear |
| Heath *et al.* (2001) [46] | + | N/A | Yes | Yes | N/A | Yes | Yes | Yes | Yes | Unclear | | Yes | Yes | Yes | No | Yes |
| Helman *et al.* (1987) [47] | - | N/A | Yes | Yes | N/A | Yes | No | No | No | No | | No | Unclear | Unclear | No | Unclear |
| Houston *et al.* (1997) [48] | Ø | N/A | Yes | Yes | N/A | Yes | No | N/A | Yes | Unclear | | Yes | Yes | Yes | Yes | Yes |
| Hua *et al.* (2001) [49] | Ø | N/A | Yes | Yes | N/A | Yes | Unclear | Yes | No | Unclear | | No | Unclear | Yes | No | Yes |
| Hunt *et al.* (1995) [50]. | Ø | Unclear | Yes | Yes | Yes | Yes | Unclear | Yes | No | Unclear | | Yes | Yes | Yes | Yes | Unclear |
| Hunt *et al.* (1999) [51]. | Ø | Unclear | Yes | Yes | Yes | Yes | No | Unclear | No | Unclear | | Yes | Yes | Yes | No | Unclear |
| Kato *et al.* (2000) [52]. | Ø | N/A | Yes | Yes | N/A | Yes | Yes | N/A | Yes | | Unclear | Yes | Yes | Yes | No | Yes |

**Table S3.** *Cont.*

| **Author** | **Quality Rating** | **Relevance Q1** | **Relevance Q2** | **Relevance Q3** | **Relevance Q4** | **Validity Q1** | | **Validity Q2** | | **Validity Q3** | **Validity Q4** | **Validity Q5** | **Validity Q6** | **Validity Q7** | **Validity Q8** | **Validity Q9** | **Validity Q10** |  |
| --- | --- | --- | --- | --- | --- | --- | --- | --- | --- | --- | --- | --- | --- | --- | --- | --- | --- | --- |
| Kim *et al.* (2012) [53]. | Ø | N/A | Yes | Yes | N/A | Yes | | Unclear | | No | No | Unclear | Yes | Unclear | Yes | Yes | Yes |  |
| Leonard *et al.* (2014) [54] | Ø | N/A | Yes | Yes | N/A | Yes | | No | | N/A | Yes | Unclear | Yes | Yes | Yes | Yes | Yes |  |
| Levin *et al.* (1986) [55] | - | N/A | Yes | Yes | N/A | Yes | | No | | Unclear | No | Unclear | No | Unclear | Unclear | No | Yes |  |
| Ley *et al.* (2014) [56] | Ø | N/A | Yes | Yes | N/A | Yes | | No | | Yes | N/A | Unclear | Yes | Yes | Yes | Yes | Yes |  |
| Locong *et al.* (1986) [57] | - | N/A | Yes | Yes | N/A | Yes | | No | | No | No | Unclear | Unclear | Unclear | Unclear | No | Unclear |  |
| Lyle *et al.* (1992) [58] | Ø | Yes | Yes | Yes | Yes | Yes | | No | | Yes | Yes | Unclear | Yes | Yes | Yes | No | No |  |
| Milman *et al.* (2004) [59] | Ø | N/A | Yes | Yes | N/A | Yes | | Unclear | | Unclear | Yes | Unclear | Yes | Yes | Yes | Yes | Yes |  |
| Noakes *et al.* (2005) [60] | + | Yes | Yes | Yes | Yes | Yes | | Yes | | Yes | Yes | Unclear | Yes | Yes | Yes | Yes | Yes |  |
| Patterson *et al.* (2001) [61] | Ø | N/A | Yes | Yes | N/A | Yes | | No | | Yes | N/A | Unclear | Yes | Yes | Yes | Yes | Unclear |  |
| Pynaert *et al.* (2007) [62] | + | N/A | Yes | Yes | N/A | Yes | | Yes | | Yes | Yes | Unclear | Yes | Yes | Yes | Yes | Yes |  |
| Reddy *et al.* (1990) [64] | Ø | N/A | Yes | Yes | N/A | Yes | | No | | No | N/A | Unclear | Yes | No | Yes | Unclear | Unclear |  |
| Rigas *et al.* (2014) [63] | Ø | N/A | Yes | Yes | N/A | Yes | | No | | N/A | Yes | No | Unclear | Unclear | Yes | Unclear | Yes |  |
| Schuepbach *et al.* (2011) [65] | Ø | N/A | Yes | Yes | N/A | Yes | | No | | N/A | Yes | Unclear | Yes | No | Yes | Yes | Unclear |  |
| Seaverson *et al.* (2007) [66] | + | N/A | Yes | Yes | N/A | Yes | | Yes | | Yes | Yes | Unclear | Yes | Yes | Yes | Yes | Yes |  |
| Snyder *et al.* (1989) [67] | Ø | N/A | Yes | Yes | N/A | Yes | | No | | Yes | No | Unclear | Yes | Yes | Yes | No | Yes |  |
| Tetens *et al.* (2007) [14] | + | Yes | Yes | Yes | Yes | Yes | | Yes | | Yes | No | Yes | Yes | Yes | Yes | Yes | Unclear |  |
| Thomson *et al.* (2011) [68] | Ø | N/A | Yes | Yes | N/A | | Yes | | Yes | No | Yes | Unclear | Yes | Yes | Yes | Yes | Yes | |
| Vaquero *et al.* (2004) [69] | Ø | N/A | Yes | Yes | N/A | | Yes | | Yes | No | N/A | Unclear | Yes | Yes | Yes | Yes | Yes | |
| Wells *et al.* (2003) [70] | Ø | Yes | Yes | Yes | Yes | | Yes | | Yes | Yes | Yes | Unclear | Yes | Yes | Yes | Yes | No | |

**Table S3.** *Cont.*

| **Author** | **Quality Rating** | **Relevance Q1** | **Relevance Q2** | **Relevance Q3** | **Relevance Q4** | **Validity Q1** | **Validity Q2** | **Validity Q3** | **Validity Q4** | **Validity Q5** | **Validity Q6** | **Validity Q7** | **Validity Q8** | **Validity Q9** | **Validity Q10** |
| --- | --- | --- | --- | --- | --- | --- | --- | --- | --- | --- | --- | --- | --- | --- | --- |
| Wilson *et al.* (1999) [71] | Ø | N/A | Yes | Yes | N/A | Yes | No | Yes | No | Unclear | Yes | Yes | Yes | Unclear | Yes |
| Wittenbecher *et al.* (2015) [72] | Ø | N/A | Yes | Yes | N/A | Yes | No | No | Yes | Unclear | Yes | Yes | Yes | Yes | Yes |
| Worthington-Roberts *et al.* (1988) [73] | Ø | N/A | Yes | Yes | N/A | Yes | No | Yes | No | Unclear | Yes | Yes | Yes | No | Unclear |
| Yokoi *et al.* (1994) [74] | + | N/A | Yes | Yes | N/A | Yes | Yes | N/A | Yes | Unclear | Yes | Yes | Yes | No | Yes |
| Yokoi *et al.* (2007) [75] | Ø | N/A | Yes | Yes | N/A | Yes | Yes | Unclear | Unclear | No | Yes | Yes | Yes | Yes | Yes |

Relevance Questions:

1. Would implementing the studied intervention or procedure result in improved outcomes for the patients/clients/population group?
2. Did the authors study an outcome (dependent variable) or topic that the patients/clients/population group would care about?
3. Is the focus of the intervention or procedure (independent variable) or topic of study a common issue of concern to dietetics practice?
4. Is the intervention or procedure feasible?

Validity Questions:

1. Was the research question clearly stated?
2. Was the selection of study subjects/patients free from bias?
3. Were study groups comparable?
4. Was method of handling withdrawals described?
5. Was blinding used to prevent introduction of bias?
6. Were intervention/therapeutic regimens/exposure factor or procedure and any comparison(s) described in detail? Were intervening factors described?
7. Were outcomes clearly defined and the measurements valid and reliable?
8. Was the statistical analysis appropriate for the study design and type of outcome indicators?
9. Are conclusions supported by results with biases and limitations taken into consideration?
10. Is bias due to study funding or sponsorship unlikely?

Quality Rating:

(−) Negative

(Ø) Neutral

(+) Positive
